# Surgical Sketches

**Published:** 1853-01

**Authors:** 


*Surgical Sketches.* By Prof. Horner.—*Wounds Involving Bones.*—When the large cylindrical bones were broken, our resort was, for the most part, amputation; we had but few cases, the result of which encouraged us to attempt the saving of others. Out of many cases of fractured os humeri and os femoris which I saw, a majority of the patients died, or were compelled to submit finally to amputation; there being but few instances of perfect recovery from such accidents. The latter circumstance was partly attributable to the largeness of the English musket balls, which almost always produced an extensively comminuted fracture when they struck a hard bone. Amongst the instances of recovery, where the thigh bone was broken, was that of Lieut Colonel McNiel of the 11th Infantry, and Captain Cilley of the same regiment, and a few privates of other corps.

Col. McNeil was wounded at Bridgewater by a ball which passed through the thigh, somewhat above the patella, and under the tendon of the extensor muscles, breaking in its course some portion of the condyles of the os femoris. The contusion was great. Much inflammation and suppuration followed, but he finally recovered with a joint somewhat stiff. He was a man of vigorous, healthy constitution, and stood more than six feet in height. The probability is that the synovial cavity was not opened; he was attended by another surgeon.

Captain Cilley had his thigh comminuted near the middle by a ball; his attending Surgeon, Dr. Trowbridge,* made four incisions into the thigh, so as to afford room for the discharge of the fragments; they were passed out in a few weeks of inflammation and suppuration, which extended but little beyond the immediate region of injury. A rapid recovery with a useful leg followed, though the length of the latter was reduced three inches.

In the cases of recovery which came under my observation, the thigh bone was broken somewhere in its lower half; this fact corresponds with the testimony of those surgeons who have had the greatest experience in gun shot wounds.† A short time after a gun shot fracture of a large bone, the limb became highly inflamed, and swollen to twice its natural size; sinuses, which opened into the wound, were the almost invariable consequences of this inflammation; after they had existed for a time, the patient's health was destroyed; he became afflicted with

*Boston Med. and Surg. Jour. 1838, p. 341.

†See Thomson's Reports of Military Observations in Belgium.

diarrhœa and hectic fever, which were very frequently fatal.

During the heat of the summer our large amputations were very unsuccessful, so much so, as almost to discourage us from performing them; many of the recent stumps mortified; in others that did not, the patient gradually sank into the arms of death. In the latter I observed there was great retraction of the muscles, and frequently the periosteum separated from the bone, for a considerable distance up. Mortification of the extremities from gun shot wounds, was very common at this period; a variety of tonic and other means were adopted to arrest its progress, and amongst the latter amputation;* nothing succeeded.

Wounds attended with much discharge were frequently infested with maggots, the ova of which were deposited by flies (Musca carnaria) in the bed clothes and dressings. This accident will probably be attributed to negligence, but the most diligent attention from nurses could not prevent it. It was a most serious evil, and frequently involved the life of the patient from the irritation produced.†

It was marvellous to see how deeply these animalcules would work their way into wounds, producing in some instances, as complete a dissection of the muscles, as if it had been performed by the knife. I learned on this occasion from a common soldier that the expressed juice of the elder bark, (Sambucus nigra,) sprinkled on the dressing and bed of the patient, would keep the flies away; and on trial I found it to be so. The suggestion was made in the case of a gallant artilleryman from Virginia, John Horton, in Captain Ritchie's company, and who had suffered the loss of both legs from a cannon shot, followed by amputation. The interest felt in him induced me to place a special nurse to prevent the approach of the flies. He was saved from the latter accident, but died in a few days, from his wounds.

*Wounds of Thorax.* Several recoveries from wounds through the thorax took place. Amongst them were

---

*See Larry, (Memoirs of Military Surgery,) who recommends this prRctice.

†An analogous fact is quoted from Alanson by J. Bell.

Lieutenant Colonel Trimble, afterwards in the United States Senate from Ohio; and an Ensign of the 19th infantry. Bleeding to a very great extent, was used in all these cases. Lieut. Col. Trimble received a ball near the junction of the 5th costal cartilage with the sternum; the ball penetrated the lung and emerged from the back near the angle of the 5th rib. The hemhorrhage from the lung by the mouth was most copious, and at each expiration blood poured freely from the external wounds, which of course implied a large collection of it in the pleura. Dyspnœa, feeble pulse, and cold extremities, left him apparently but little chance of life, but on the fifth day the symptoms mitigated. He was bled in the subsequent twenty-two days, six times by his attending surgeon, Dr. Amos Trowbridge,* and finally recovered. While a member of the Senate of the United States, he died at Washington in 1822, from a pulmonary affection, brought on by exposure in the preceding season.

*Wounds of the Abdomen.* Wounds of the abdomen, and its contents were, in several instances, recovered from; amongst such were Lieutenant Cisna, of the 19th infantry, and a Sergeant White. In the first, the ball entered in the anterior part of his belly, below the naval, and came out near the spine; about six weeks afterwards a button was discharged from the wound, near the spine, which had been carried in along with the ball.

*Amputations of the Leg.* I did not see a case of recovery where amputation had been performed on both legs, or on both thighs, in one subject; but I saw a few recoveries *where* a leg and an arm, or two arms had been resected. Several double accidents of this kind happened during the seige of Fort Erie. In one instance, a cannon ball entered a hospital, and ranged along the feet of a row of bedsteads, carrying away several legs in its course. In another instance, a young rifleman of eighteen, belonging to Captain Irvine's company, had both his arms taken off above the elbow joint, by a cannon shot. The ball being nearly spent, he then started in pursuit of it, not appalled by what had happened, but continuing to kick it as an enemy until its motion gradually subsided. His gallantry and coolness on this occasion procured him the notice of several distinguished officers. I saw one case of recovery

* For details, see Bost. Med. and Surg. Jour. for 1838, p. 344.

from amputation at the shoulder joint. This operation was performed by Dr. Gales, of the 23d infantry, whose services and intrepidity gained a high distinction.

*Lacerated Wounds.* Some cures, from extensively lacerated wounds, took place; amongst them, J. King, a private in Captain Biddle's company of artillery. He had the whole posterior part of his thigh, from the buttock to the ham, torn up by the fragment of a shell, and the muscles laid bare, as in a dissection. This extensive wound, in which there was a great loss of integuments, cicatrized and got nearly well in three or four months. A private in the Infantry already alluded to, was scalped in a circular line, just above the tips of his ears. Granulations arose from the exposed surface of bone and pericranium, in a few days, and cicatrized very kindly ; the discharge of pus was, for a long time, very considerable.—*Medical Examiner.*

